# Dog Bite Injuries: Primary and Secondary Emergency Department Presentations—A Retrospective Cohort Study

**DOI:** 10.1155/2013/393176

**Published:** 2013-09-24

**Authors:** Carmen A. Pfortmueller, Anastasios Efeoglou, Hansjakob Furrer, Aristomenis K. Exadaktylos

**Affiliations:** ^1^Department of General Internal Medicine, Bern University Hospital, University of Bern, 3010 Bern, Switzerland; ^2^Department of Emergency Medicine, Bern University Hospital, University of Bern, 3010 Bern, Switzerland; ^3^Department of Visceral Surgery, Basel Bruderholz Cantonal Hospital, 4059 Basel, Switzerland; ^4^Department of Infectious Diseases, Bern University Hospital, University of Bern, 3010 Bern, Switzerland

## Abstract

Dog bites in humans are a complex problem, embracing both public health and animal welfare. The primary aim of this study is to examine primary and secondary presentations related to dog bite injuries in adults. *Methods*. We retrospectively assessed all adult patients admitted with a dog bite injury to the Emergency Department of Bern University Hospital. *Results*. A total of 431 patients were eligible for the study. Forty-nine (11.4%) of all patients were admitted with secondary presentations. Bites to the hands were most common (177, 41.1%). All patients (47, 100%) with secondary presentations were admitted because of signs of infection. The median time since the dog bite was 3.8 days (SD 3.9, range 1–21). Thirty-one patients had already been treated with antibiotic; coamoxicillin was the most common primary antibiotic therapy (27/47 patients, 57.4%). Patients with injuries to the hand were at increased risk of secondary presentations (OR 2.08, 95% CI 1.21–3.55, *P* < 0.006). *Conclusion*. Dog bite injuries to the hands are a major problem. They often lead to infectious complications. Immediate antibiotic therapy should carefully be evaluated for each patient.

## 1. Introduction

The close association between humans and domesticated dogs began at least 12,000 years ago [1]. However, the dog is a former wild animal and retains its instincts, including behaviour that may lead to attacks on man [1]. Dog bites in humans are a complex problem, embracing both public health and animal welfare [2–4]. Dog bites are placed worldwide among the top 12 causes of nonfatal injuries [5].

It has been estimated that the risk of being bitten by a domestic animal during a lifetime is about 50%, of which dog bites account for 80–90% [6]. But only 10–50% of all dog bite injuries are reported to medical services [3]. In the USA, approximately 333,687 dog bite injuries are treated annually in emergency departments [1]. Even though dog bite injuries are frequent, they are preventable [2].

In 2011, about 371,000 dogs lived in Switzerland, and a dog was present in 12% of all Swiss households [7]. Since 2006, all dog bite injuries in Switzerland have to be reported to the Federal Veterinary Agency and labelled as such in medical records [8]. In 2009, a total of 2843 dog bite injuries to humans were reported [9]. It is not known how many of these needed medical attention. 

There have been several studies that focus on dog bite injuries and their epidemiology, especially in the 1990s. Several preventive and legal measurements were then implemented to reduce the incidence of dog bites, but current studies are scarce. Moreover, most studies focus on children, as the incidence is particularly high in this population [10]. Dog bite injuries in adults have been less well studied. Our study therefore focuses on emergency department admissions related to dog bites in adults. The aim of this study is to examine primary (within 24 hours after a dog bite) and secondary presentations (presentation > 24 hours after a dog bite) related to dog bite injuries in adults.

## 2. Material and Methods

### 2.1. Setting

Our ED is the only Level I centre in a catchment area serving about 1.8 million people, and it treats more than 35,000 cases per year. 

### 2.2. Data Collection and Retrospective Survey

Our retrospective data analysis comprised adult (≥16 years) patients admitted to our emergency department between 1 January 2000 and 31 October 2012 in relation to a dog bite. All patients presenting to the ED with a dog bite during the study period were initially eligible for study inclusion. They were identified using the appropriate search string in the diagnosis or medical history field of our computerised patient database (Qualicare Office, medical database software, Qualidoc AG, Inselspital Bern, Switzerland). The following clinical data were extracted from medical records: admission date, type of dog, relationship to the dog, environment of the bite, site of injury, depth of injury, initial treatment, hospitalisation, and secondary presentations. A secondary presentation was defined by the study team as presentation > 24 hours after a dog bite. In patients with secondary presentations, the following data were additionally assessed: time since dog bite, institution of primary treatment (if any), type of primary treatment (if any), and change/start of treatment on admission to our ED. Demographic data such as gender and age were also assessed. Elderly patients were defined as patients equal to or older than 65 years of age. The size of the dog was categorised as small, medium, large, or extra large [11, 12]. If a patient was bitten several times, the case was categorized as “multiple”. If depth of injury was not explicitly mentioned in the medical records, this was estimated by the study team. Depth of injury was then split into three categories: superficial (0.1–0.2 cm), medium (0.3–1 cm), or deep (more than 1 cm). Patients with duplicated records (*n* = 4), a dog bite in their past medical history not related to the presentation (*n* = 5), and incomplete records (*n* = 10) (injury site and depth of dog bite were not extractable from the medical history) were excluded from the analysis. 

### 2.3. Statistical Analysis

All statistical analyses were performed with the SPSS 20.0 statistical analysis program (SPSS Inc; Chicago, IL). The data were summarised using descriptive statistics (means and standard deviation or medians as appropriate, counts, and percentages). Differences in characteristics and outcome between patients with primary and secondary presentations were tested using chi-squared tests for categorical variables and the Kruskal-Wallis ANOVA for interval and ordinal variables. Post-hoc testing was performed using the Mann-Whitney *U* test. Multivariable logistic regression was used to identify predictors for secondary presentations and hospitalisation. The predefined variables added to the model were gender, age, localisation of injury, injury depth, and need for surgical treatment, need for hospitalisation. All *P* values were two tailed and at a level of significance of 0.05.

## 3. Results

**Figure 1:**
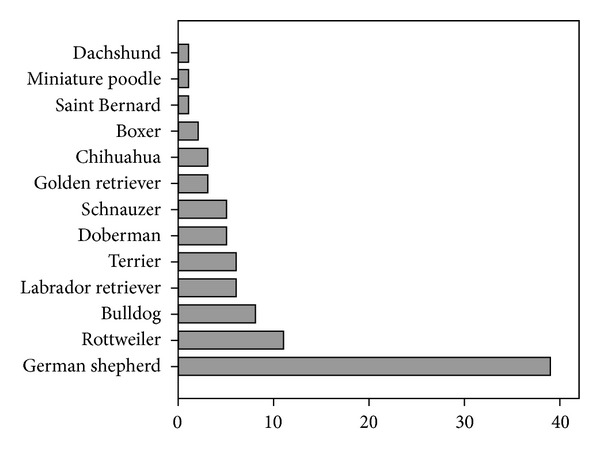
Dog types (21.1% known, 78.9% unknown).

Of 350,000 ED visits over an eleven-year study period, a total of 431 patients were eligible for the study.  Table 1 lists the patient characteristics. Forty-seven (11.4%) of all patients were admitted with secondary presentations, whereas 382 (88.6%) of presentations were primary. The dog breed was unknown in 340 (78.9%) cases. The most commonly known dog breed was German shepherd (39, 9.0%). See Figure 1. There was no correlation between dog size or type and depth of injury (*P* < 0.59 and *P* < 0.83, resp.). Bites to the hands were most common (177, 41.1%). Superficial dog bite injuries were most common (340, 78.9%). They were associated with injuries to the face (*P* < 0.005) and the lower extremity (*P* < 0.001). Deep injuries were correlated with injures to the upper extremity (*P* < 0.001). There was no difference in the depth of injury between patients with single and those with multiple bites (*P* < 0.95). There was no correlation between dog size and injury type (*P* < 0.17). Injuries to the hand were more common in the older cohort (*P* < 0.03), whereas injuries to the lower extremity were more frequent in the younger population (*P* ≤ 0.23). Elderly patients more often needed operative treatment (*P* < 0.006) and were more often hospitalized (*P* < 0.001).

**Figure 2:**
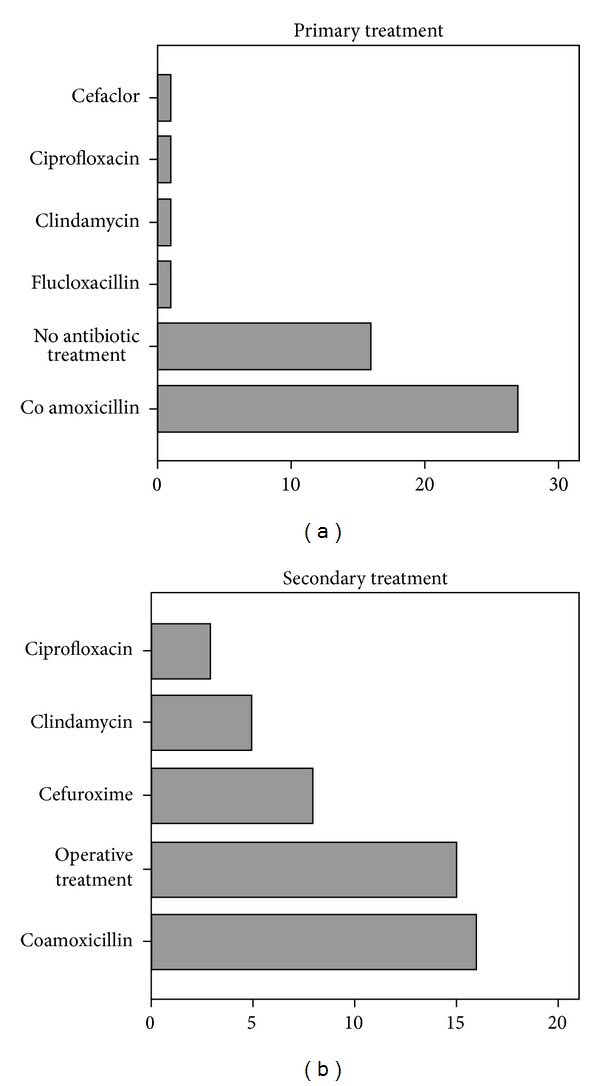
Treatment of patients with secondary presentations: primary (a) and secondary (b) antibiotic therapy.

Table 2 summarizes the characteristics of patients with secondary presentations. For the 47 (11.4%) patients with secondary presentations, the median time since the dog bite was 3.8 days (SD 3.9, range 1–21). The time span was not associated with the depth of injury (*P* < 0.12), nor with the localisation of injury (*P* < 0.47). All these patients (47, 100%) presented because of signs of infection. Thirty-one (65.9%) were already being treated with an antibiotic when presenting to our ED; co-amoxicillin was the most common primary antibiotic (27/47 patients, 57.4%). See Figure 2 for an overview on the treatment of patients with secondary presentations. Note that 16/47 (34.0%) of all patients with secondary presentations had not yet received any antibiotic therapy and that 15 (31.9%) of the patients with secondary presentations had to be operated on. Infections occurred more frequently in patients with injuries to the hands (*P* < 0.006) and were associated with a greater need for surgical treatment (OR 10.01, 95% CI 6.91–14.84, *P* < 0.0001). Depth of injury was not associated with secondary presentations (*P* < 0.18). Age was not associated with increased risk of secondary presentations (OR 1.1, 95% CI 0.96–1.24, *P* < 0.068).

Age, injury depth, and secondary presentations were risk factors for hospitalisation (all *P* < 0.001). 

## 4. Discussion

We aimed to characterise primary and secondary presentations after dog bite injuries. 

Several studies have found that the hands are the parts of the body most frequently injured in dog bites [3, 13]. Our study shows that advancing age is associated with a higher proportion of dog bite injuries to the hands. We can only speculate about the reasons. As elderly patients may suffer from impaired sight, neurological diseases, or loss of coordination, they may be less able to interpret and react to the changes in the dog's behaviour [14]. Furthermore, their reaction time may be prolonged [14]. Therefore dog bites to the hand may be more frequent in the older population as they try to protect themselves by pushing the dog away. In contrast, younger people may interact differently with dogs [15], by playing with them roughly, or they may even attempt to separate two fighting dogs [6]. This makes them vulnerable to injuries to other body regions, such as the lower extremity. 

Our study confirms that patients with dog bite injuries to the hands are especially vulnerable to developing secondary infectious complications after a dog bite [3, 16]. According to Rothe et al., the hands—with their close topographical relation to bradytrophic tissue such as tendons to the skin surface—are especially prone to develop infectious complications [16]. Moreover, as there are no natural anatomical barriers, an infection to the hand can easily spread along these structures [16].

The failure to implement antibiotic treatment immediately after a dog bite injury in our study was not associated with an increased risk of subsequent secondary presentations related to infection. It is not clear whether prophylactic or preemptive antibiotic treatment should be given to all patients with dog bite injuries [3, 16]. Some authors have found that patients with bites may benefit from immediate antibiotic therapy [17], whereas others did not find any benefit [18]. We opt for a differentiated approach and propose 3–5 day antibiotic therapy with amoxicillin-clavunate treatment in those patients with higher infection risk, such as those with deep injuries with crushed tissues, injuries to the hands, patients who present to medical care more than 24 hours after the bite, and immunocompromised hosts [3, 19]. 

Interestingly, no correlation was found between the depth of injury and secondary presentations related to infectious complications. One would have expected that large wounds would more often be infected, as they are more difficult to clean efficiently with disinfectant detergents and are more difficult to reach with antibiotics. Nevertheless, we found no significant relationship between depth of injury and infectious complications. 

Even though there was no significant relationship between age and secondary presentations related to infection in our study, we believe that there may be a correlation that was not detected in our relatively small study population. Thus, elderly people are more vulnerable to suffer from any kind of infection due to decreases in the efficiency of the immune system with advancing age [20]. This may be enhanced by increased rates of immunosuppressive medication, neoplastic disease, or malnutrition. Therefore, dog bites in the elderly may lead to increased numbers of infections. 

In this study, age was associated with increased risk for hospitalisation. This may have several reasons. Firstly, the elderly suffer from various types of medical conditions [14] and an additional injury may make them unable to care for themselves at home. Secondly, elderly people often live alone, as they are widowed or divorced [21], and therefore they do not have appropriate support at home to manage alone. 

## 5. Limitations

Our findings have to be assessed with some caution, as some parameters were not available for the whole study population and our sample size is quite small. Data on dog size and dog ownership have to be read with special caution. Furthermore, we unfortunately have no data on the length of antibiotic treatment, the range of bacteria, the aesthetic outcome, and reasons for hospitalisation, as this data is not retrospectively assessable. Therefore, no conclusion on long-term outcome and treatment can be drawn from this study. Additionally, as this is a single centre study, our data are probably not generalizable for the whole of Switzerland. As information in our medical database is presented in a narrative way, no guarantee of complete or correct reporting can be given, and bias is possible. 

Furthermore, as our ED only treats adults older than 15 years of age, no information on dog bite injuries and related secondary presentations in children can be given.

## 6. Conclusion

Dog bite injuries to the hands are a major problem. They often lead to infectious complications. As lack of prophylactic antibiotic therapy immediately after a dog bite may be associated with increased risk of secondary presentations related to infection in high risk patients, this should be carefully considered for each patient. Further studies are needed with larger cohorts and more detailed data acquisition and follow-up. 

## Figures and Tables

**Table 1:** Patient's characteristics.

	*N*	%
*N* total	431	100
Demographic data		
Male/female	258/173	59.9/40.1
Age (years) median (range)	36 (16–87)	
<65	391	90.7
>65	40	9.3
Size of dog		
Small	10	2.3
Medium	40	9.3
Large	13	3
Extra large	28	6.5
Unknown	340	78.9
Relationship to the dog		
Friendly	83	19.3
Unfriendly	101	23.4
Unknown	247	57.3
Localisation of accident		
Indoors	29	6.7
Outdoors	130	30.2
Unknown	272	63.1
Localisation of injury		
Face	40	9.3
Hand	177	41.1
Upper extremity	55	12.8
Lower extremity	133	30.9
Buttocks	8	1.9
Genitals	3	0.7
Multiple	13	3
Depth of injury		
Superficial	340	78.9
Median	68	15.8
Deep	23	5.3
X-ray (total)	93	21.6
Pathological findings	16	17.3
Blood sample	179	41.5
Mean CRP level (SD, range)	19 (40.7, 3–240)	
Mean Lc count (SD, range)	8.2 (4.2, 4.4–21.8)	
Treatment		
Antibiotics	304	70.4
Operation	18	4.2
None	109	25.4
Hospitalisation	26	6
Secondary presentations	49	11.4

**Table 2:** Secondary presentations.

	*N* (%)	*N* (%)	*P* value
	Primary presentation	Secondary presentation	
Mean time until second presentation (days) (SD, range)		3.8 (3.9, 1–21)	
Reason for secondary presentation (infection)		47 (100)	
Sex (male/female)	229/153	27/20	0.91
Size of dog			0.11
Localisation of injury			
Face	40 (10.4)	2 (4.1)	0.15
Hand	148 (38.7)	28 (59.1)	0.006
Upper extremity	50 (13.1)	5 (10.2)	0.56
Lower extremity	120 (31.4)	12 (26.5)	0.48
Buttocks	8 (2.1)	0 (0)	0.31
Genitals	3 (0.8)	0 (0)	0.53
Multiple	13 (3.4)	0 (0)	0.19
Depth of injury			0.18
Location of primary presentation			
Emergency department		8 (18.4)	
General practitioner		8 (16.3)	
Self-treatment		2 (4.1)	
No primary presentation		29 (61.2)	
Primary antibiotic treatment		31 (65.9)	
Blood sample			
Median CRP level (unit)	7.1	24.4	0.0001
Median lc count (unit)	3	9.3	0.0001
X-ray	74 (19.3)	19 (38.7)	0.002
Pathological findings	13 (3.4)	3 (6.1)	0.34
Secondary treatment			
Change/initiation of antibiotics		22 (46.9)	
Operation		15 (30.6)	
Continued antibiotics		10 (22.4)	
Hospitalisation	14 (3.7)	12 (24.5)	0.0001
